# Toxicity of Necrostatin-1 in Parkinson’s Disease Models

**DOI:** 10.3390/antiox9060524

**Published:** 2020-06-15

**Authors:** Eva Alegre-Cortés, Alicia Muriel-González, Saray Canales-Cortés, Elisabet Uribe-Carretero, Guadalupe Martínez-Chacón, Ana Aiastui, Adolfo López de Munain, Mireia Niso-Santano, Rosa A. Gonzalez-Polo, José M. Fuentes, Sokhna M. S. Yakhine-Diop

**Affiliations:** 1 Department Bioquímica y Biología Molecular y Genética, Facultad de Enfermería y Terapia Ocupacional, Universidad de Extremadura Avda de la Universidad s/n, 10003 Cáceres, Spain; evalegrec@unex.es (E.A.-C.); agonzalesp@alumnos.unex.es (A.M.-G.); sacanalesc@unex.es (S.C.-C.); euribec@unex.es (E.U.-C.); guadalupemc@unex.es (G.M.-C.); mnisosan@unex.es (M.N.-S.); rosapolo@unex.es (R.A.G.-P.); 2 Centro de Investigación Biomédica en Red de Enfermedades Neurodegenerativas (CIBERNED), 28049 Madrid, Spain; 3 Instituto Universitario de Investigación Biosanitaria de Extremadura (INUBE), 10003 Cáceres, Spain; 4 Cell Culture Plataform, Donostia University Hospital, 20014 San Sebastián, Spain; ANA.AIASTUI@biodonostia.org; 5 Neuroscience Area of Biodonostia Health Research Institute, Donostia University Hospital, 20014 San Sebastián, Spain; ADOLFOJOSE.LOPEZDEMUNAINARREGUI@osakidetza.eus; 6 Department of Neurology, Donostia University Hospital, 20014 San Sebastian, Spain; 7 Ilundain Fundazioa, 20014 San Sebastian, Spain; 8 Department of Neurosciences, University of the Basque Country UPV-EHU, 20014 San Sebastián, Spain

**Keywords:** mitochondria, mitophagy, MLKL, necroptosis, RIP, rotenone

## Abstract

Parkinson’s disease (PD) is a neurodegenerative disorder that is characterized by the loss of dopaminergic neurons in the substantia nigra pars compacta. This neuronal loss, inherent to age, is related to exposure to environmental toxins and/or a genetic predisposition. PD-induced cell death has been studied thoroughly, but its characterization remains elusive. To date, several types of cell death, including apoptosis, autophagy-induced cell death, and necrosis, have been implicated in PD progression. In this study, we evaluated necroptosis, which is a programmed type of necrosis, in primary fibroblasts from PD patients with and without the G2019S *leucine-rich repeat kinase 2* (*LRRK2)* mutation and in rotenone-treated cells (SH-SY5Y and fibroblasts). The results showed that programmed necrosis was not activated in the cells of PD patients, but it was activated in cells exposed to rotenone. Necrostatin-1 (Nec-1), an inhibitor of the necroptosis pathway, prevented rotenone-induced necroptosis in PD models. However, Nec-1 affected mitochondrial morphology and failed to protect mitochondria against rotenone toxicity. Therefore, despite the inhibition of rotenone-mediated necroptosis, PD models were susceptible to the effects of both Nec-1 and rotenone.

## 1. Introduction

Parkinson’s disease (PD) is a neurodegenerative disorder characterized by progressive neuronal loss in various regions of the central nervous system. The most prominent aspect is the loss of dopaminergic neurons in the *substantia nigra pars compacta* [1]. This extensive neuronal death triggers motor and non-motor clinical features which uncover the PD pathogenesis that has probably been underway for a long time. The pathogenesis of PD is very complex and has been reported to be related to gene mutations and/or to long-term exposure to stressor agents throughout an individual’s life. PD is classified into genetic and idiopathic forms, with both leading to the degeneration of neurons. It is still not clear what renders neurons susceptible to death. Studies focusing on toxin-induced PD models (paraquat [2,3], rotenone, 6-hydroxydopamin (6-OHDA), 1-methyl-4-phenylpyridinium iodide (MPP^+^) [4]) as well as on genetic PD cases (LRRK2 [5,6], PTEN-induced putative kinase 1 (PINK1) [7,8], DJ1, α-synuclein (SNCA)) point out that, at first glance, cell death in PD appears to be related to mitochondrial dysfunction, reactive oxygen species (ROS) generation, cytochrome c release, activation of mitogen-activated protein kinase (MAPK) pathways, autophagy impairment, and/or disruption of calcium homeostasis. In general, when these signaling cascades fail, the effort required to sustain a protective effect leads cells to undergo autophagy-induced cell death [4], apoptosis [9], and/or necrotic cell death [10].

Necroptosis is a programmed type of necrosis that develops in response to the activation of two members of the receptor-interacting protein (RIP) family, RIP1 and RIP3. Both are serine/threonine kinase proteins [11] that interact with each other via their RIP homotypic interaction motifs (RHIM). Indeed, RIP1 activation requires autophosphorylation, and thereafter, phosphorylates the RIP3 protein [12]. Beyond phosphorylation, RIP1 and RIP3 form a complex called a necrosome [13] which activates the mixed lineage kinase domain-like (MLKL) protein, a RIP3 substrate that is critical for plasma membrane rupture [14]. In fact, upon phosphorylation by RIP3, MLKL forms oligomers that translocate to the plasma membrane and promote either ion influx (calcium and sodium) or pore formation, which is responsible for a loss in membrane integrity [13]. Such membrane disruption triggers inflammation through the release of cell contents and, eventually, leads to cell death. The involvement of necroptosis in PD has been demonstrated in animals injected with 1-methyl-4-phenyl-1,2,3,6-tetrahydropyridine (MPTP) [15], with 6-OHDA and in postmortem PD midbrain samples [16] through an increase in MLKL phosphorylation. In addition, necrostatin-1 (Nec-1), an inhibitor of the necroptotic pathway, exerts a neuroprotective effect on 6-OHDA-treated pheochromocytoma (PC12) cells [17], on MPTP-treated mice [15,18], and prevents neurite degeneration in 6-OHDA mesencephalic neurons [16]. Although non-apoptotic death induced by MPP^+^ was shown to be inhibited by Nec-1 in differentiated SH-SY5Y cells, this was not classified as necroptosis [10].

In this study, we used primary fibroblasts from PD patients with or without the G2019S *LRRK2* mutation to characterize the necroptosis pathway. Moreover, we assessed the oxidative stress-inducing effect of rotenone in PD models. Interestingly, protein executioners of necroptosis were expressed in PD patients, but were only activated under rotenone treatment. Nec-1 totally abolished rotenone-induced necroptosis but did not prevent rotenone toxicity.

## 2. Materials and Methods

### 2.1. PD Models

Experiments were carried out with fibroblasts from healthy subjects (Control, Co.) and patients with two forms of PD, namely, idiopathic PD (IPD, PD patients without the G2019S *LRRK2* mutation), and GS (PD patients with the G2019S *LRRK2* mutation). All subjects gave written informed consent in accordance with the Declaration of Helsinki and in agreement with the Comité Ético de Investigación Clínica del Área Sanitaria de Gipuzkoa (Ethical protocol code ALM-LRRK2-2016-01). In parallel, neuroblastoma cells, SH-SY5Y, were used to confirm the results obtained with human fibroblasts (HFs). Both cell types were maintained in Dulbecco’s Modified Eagle’s Medium (DMEM, Sigma-Aldrich, D6546) supplemented with 1% L-glutamine (Sigma-Aldrich, G7513), antibiotics (HyClone Laboratories, SV30010), and 10% fetal bovine serum (FBS, Sigma-Aldrich, F7524). Cells were seeded at 35,000 cells/mL for HFs and 100,000 cells/mL for SH-SY5Y. They were treated with carbonyl cyanide 3-chlorophenylhydrazone (CCCP, Sigma-Aldrich, C2759), rotenone (Sigma-Aldrich, R8875), and/or Nec-1 (Abcam, ab141053).

### 2.2. Western Blotting Analysis

Cells were lysed in buffer containing 0.5% NP-40 (Roche, 11754599001), 100 mM Tris-HCl (pH 7.4), and 300 mM NaCl and complemented with protease and phosphatase inhibitors [19]. Proteins were loaded by SDS-gel electrophoresis, and blots were probed with antibodies against glyceraldehyde 3-phosphate dehydrogenase(GAPDH) (6C5) (Millipore, NG1740950, dilution 1:5000), MLKL (D2I6N) (N° 14993, dilution 1:2500), p-MLKL (Ser358)(D6H3V) (N° 91689, dilution 1:2500), RIP1 (D94C12, dilution 1:1000) (N° 3493), prohibitin-1 (PHB1) (N° 2426S, dilution 1:1000) and p-RIP1 (Ser166)(D1L3S) (N° 65746, dilution 1:1000) from Cell Signaling, Lon peptidase 1 (LONP1) (Proteintech, 15440-1-AP, dilution 1:1000), p-RIP3 (Ser227) (EPR9627) (Abcam, ab209384, dilution 1:8000), and translocase of outer mitochondrial membrane 20 (TOMM20) (F-10) (Santa-Cruz, sc-17764, dilution 1:1000). Western blot images were analyzed by ImageJ software.

### 2.3. Flow Cytometry Assay

Cells were preloaded with 100 nM MitoTracker^®^ Green FM (MTG, Thermo Fisher, M7514) and washed with phosphate-buffered saline (PBS) before treatment [19]. To monitor mitochondrial mass, cells were collected in FACS tubes and loaded with MTG, as previously reported [19]. For the assessment of cell death, cells were stained with annexin V-FITC (Immunostep, ANXVF-200T) for 15 min at 37 °C, and afterwards, propidium iodide (0.1 mg/mL) (PI, Sigma-Aldrich, P4170) was added to each tube to detect either the percentage of PI or annexin-positive (+) cells. Otherwise, we measured ROS levels by detecting the accumulation of superoxide with 5 μM dihydroethidium (Invitrogen, D1168). To study the mitochondrial membrane potential (MMP) and mitochondrial ROS, we used 20 nM tetramethylrhodamine, methyl ester, perchlorate (TMRM, Invitrogen™, T668), and 2 μM MitoSOX^TM^ (Invitrogen™, M36008), respectively. MitoSOX and dihydroethidium were oxidized to ethidium and emitted a red fluorescence. All stained cells were analyzed by flow cytometer (Beckman Coulter FC500-MPL).

### 2.4. Immunofluorescence Microscopy

Cells were plated on pretreated coverslips with poly-L-lysine. After rotenone treatment, cells were washed with annexin buffer 1X and incubated with annexin V-FITC (1X) for 15 min at room temperature (RT). Subsequently, 5 μL of PI was directly added to cells. Thereafter, cells were washed and incubated with annexin buffer 1X to wipe away excess dye. Annexin/PI staining was observed by in vivo immunofluorescence. Nuclei were stained with 300 nM of 4′, 6-diamidino-2-phenylindole (DAPI) (Thermo Fisher, D1306). For mitochondrial morphology, cells were fixed with 4% paraformaldehyde (PFA) and permeabilized with 0.1% Triton X-100 (Sigma-Aldrich, T9284). Once permeabilized, cells were incubated with bovine serum albumin (BSA)/PBS solution (1 mg/mL) for 1 h. Thereafter, cells were incubated for 1 h with TOMM20 (F10) (Santa-Cruz, sc-17764, dilution 1:200) and, subsequently, with Thermo Fisher Alexa Fluor^®^ 568 (A11004)-conjugated secondary antibodies for another hour at RT. Results were analyzed, and represented the proportion of cells with filamentous and damaged mitochondria for each condition. Images were visualized using an Olympus IX51 inverted microscope equipped with a DP71 camera.

### 2.5. Statistics

Data were collected and analyzed using Microsoft Excel and/or SPSS software. Statistical analyses were done using Student t and Χ^2^ tests to establish the significant differences (*p* values * < 0.05, ** ≤ 0.01, *** ≤ 0.001) between control and PD cell lines under basal and treated conditions.

## 3. Results

### 3.1. Characterization of Necroptosis in PD Models

**Figure 1:**
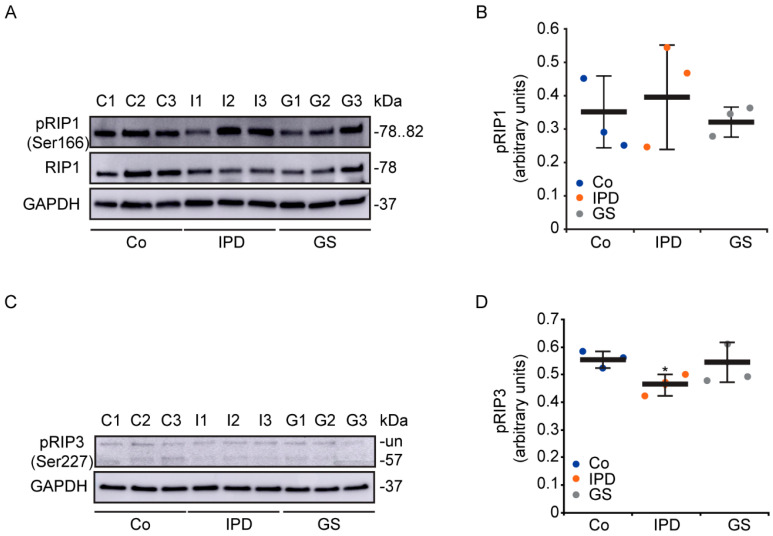
Necroptosis pathway in Parkinson’s disease (PD) patients. (**A**–**D**) Cells were maintained under basal conditions. Expression levels of p-receptor-interacting protein (RIP)1 (**A**,**B**) and p-RIP3 (**C**,**D**), which are involved in the signaling pathway of necroptosis, were determined and normalized to the loading control, GAPDH. Data are the value of the representative blot and correspond to the mean ± SD of three independent lines (* *p* < 0.05, compared to control (Co) lines). Each group consisted of three cell lines: control (C1, C2, and C3), idiopathic Parkinson’s disease (IPD: I1, I2, I3), and GS (PD patients with the G2019S *LRRK2* mutation: G1, G2, G3) groups; un: unspecified. Experiments were done at least three times.

Apoptotic [5] and necrotic [19] cell death have been described in cells from patients with genetic or sporadic PD, respectively. In this study, we characterized the necroptosis pathway in primary fibroblasts from PD patients harboring or not harboring the G2019S *LRRK2* mutation (Figure 1). Based on the phosphorylation levels of RIP1 (p-RIP1) (Figure 1A,B) and RIP3 (p-RIP3) (Figure 1C,D), the necroptosis pathway did not seem to be activated in cells from PD patients. Although there were no differences in p-RIP1 levels among the groups, p-RIP3, the substrate of p-RIP1 was significantly decreased in IPD cells and not in GS cells. Therefore, we did not observe any significant protein phosphorylation levels that could show that the necroptosis pathway was activated in PD patient cells. However, proteins responsible for necroptosis activation were found to be well-expressed in human fibroblasts.

### 3.2. Study of Necroptosis in Rotenone-Induced Models

**Figure 2:**
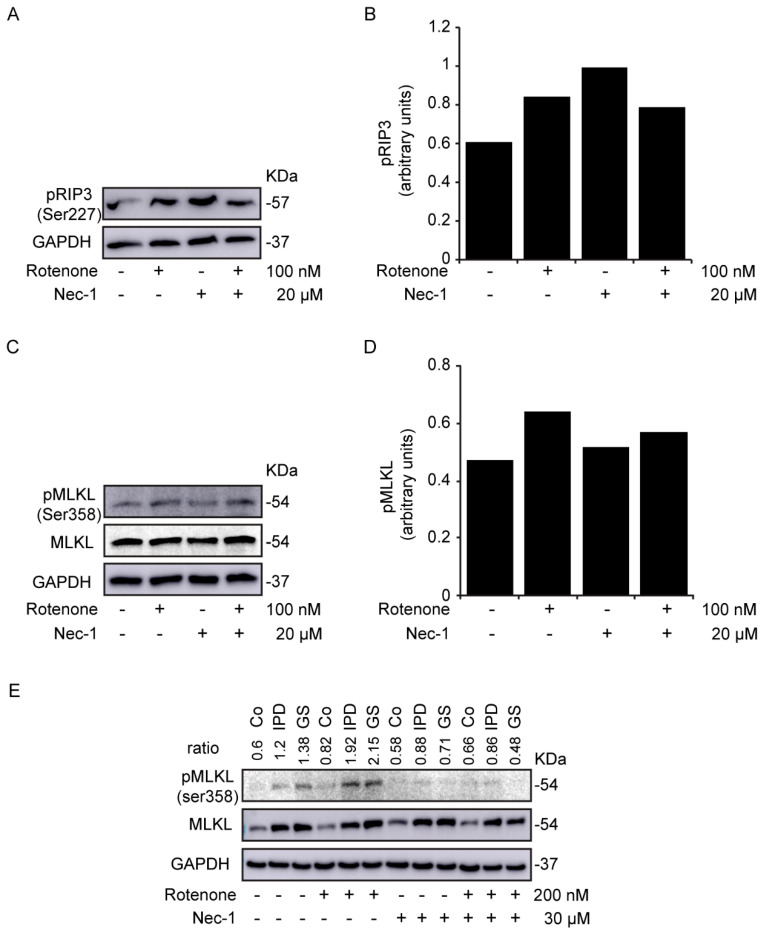
Rotenone-induced necroptosis in SH-SY5Y cells. (**A**–**E**) Cells were treated with rotenone and/or necrostatin-1 (Nec-1) for 24 h. The expression levels of p-RIP3 (**A**,**B**) and p-mixed lineage kinase domain-like (MLKL) protein (**C**,**D**) were determined in SH-SY5Y cells and normalized to the loading control, GAPDH. Data are the values of the representative blot. (**E**) is the representative blot of p-MLKL and MLKL expression levels in human fibroblasts. The ratio is the densitometry of p-MLKL/MLKL. GAPDH was used as the loading control. Experiments were conducted three times.

Cells were treated with the mitochondrial complex I inhibitor rotenone and/or Nec-1, an RIP1 inhibitor [10], for 24 h. In rotenone-treated SH-SY5Y cells, we observed an increase in the levels of p-RIP3 (Figure 2A,B) and its substrate, p-MLKL (Figure 2C,D). Similar data were mentioned for p-RIP3 in a study involving primary culture of mesencephalic neurons with much lower doses of rotenone [20]. However, while Nec-1 treatment failed to decrease the level of p-RIP3, the level of p-MLKL was reduced. As a consequence, Nec-1 inhibited rotenone-induced necroptosis. We wondered whether rotenone could activate necroptosis in the cells of PD patients. Interestingly, we noticed an enhanced level of p-MLKL with rotenone treatment (Figure 2E), which reduced with Nec-1. We inferred then that Nec-1 efficiently inhibited the activation of necroptosis by rotenone in both neuroblastoma cells and primary fibroblasts. Of note, the expression level of MLKL in the cells of PD patients was not affected by Nec-1. However, a previous study has related the upregulation of MLKL to necroptosis activation in human neurons with OPA1 mutations [18]. In our study, a different variation of MLKL was also observed during its characterization in primary fibroblasts (Figure S1), but was not significant.

### 3.3. Cellular Toxicity of Necrostatin-1

**Figure 3:**
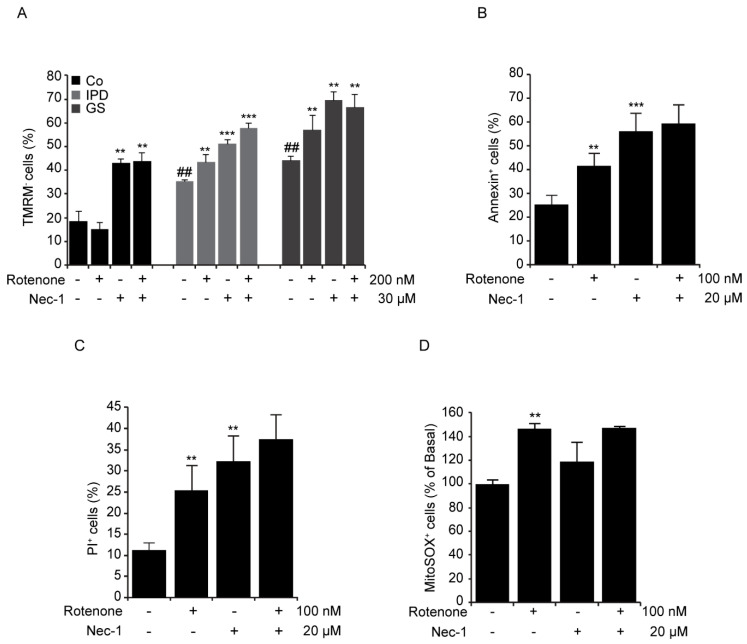
Rotenone-induced necroptosis in PD patients. (**A**–**D**) Cells were treated with rotenone and/or necrostatin-1 (Nec-1) for 24 h. (**A**) Cells were loaded with 50 nM tetramethylrhodamine, methyl ester, perchlorate (TMRM). The results represent the percentage ± SD of TMRM-negative cells detected by flow cytometry (** *p* < 0.01, *** *p* < 0.001 in comparison with basal conditions; ^##^ *p* < 0.01 in comparison to Co line), *n* = 10,000 events. (**B**,**C**) SH-SY5Y cells were costained with annexin-FITC and propidium iodide (PI). The results represent the percentage ± SD of annexin^+^ or PI^+^ cells detected by flow cytometry (** *p* < 0.01, *** *p* < 0.001 compared to basal conditions), *n* = 10,000 events. (**D**) SH-SY5Y cells were loaded with MitoSOX (2 μM). The results represent the percentage ± SD of MitoSOX^+^ cells detected by flow cytometry (** *p* < 0.01, compared to basal conditions), *n* = 10,000 events. Experiments were done three times.

Rotenone induces mitochondrial dysfunction, resulting in the generation of mitochondrial ROS and the loss of MMP [21]. A previous study showed that oxidative stress leads to necroptosis activation [22]. Given that Nec-1 abrogated rotenone-induced necroptosis, we decided to study MMP by loading cells with TMRM. While healthy mitochondria retained the dye, the damaged one did not. The percentage of TMRM-negative (-) cells was remarkably augmented following Nec-1 treatment with or without rotenone incubation (Figure 3A). Rotenone decreased MMP by 10% in the cells of PD patients. Unexpectedly, it did not affect MMP in the control line, but it exacerbated the number of annexin^+^/PI^+^ cells (data not shown) observed with fluorescence microscopy. Although Nec-1 inhibited the induction of necroptosis in rotenone-treated SH-SY5Y cells, it was not able to decrease the percentage of annexin^+^ cells (Figure 3B) and/or PI^+^ cells (Figure 3C), nor did alter the level of mitochondrial ROS produced with rotenone (Figure 3D). Taken together, rotenone induced not only necroptosis but also apoptosis [21] and/or necrosis [20] in cell cultures, as previously reported. Surprisingly, Nec-1 treatment alone resulted in an increase in annexin/PI^+^ cells and a decrease in MMP.

### 3.4. Mitochondrial Morphological Changes with Necrostatin-1

**Figure 4:**
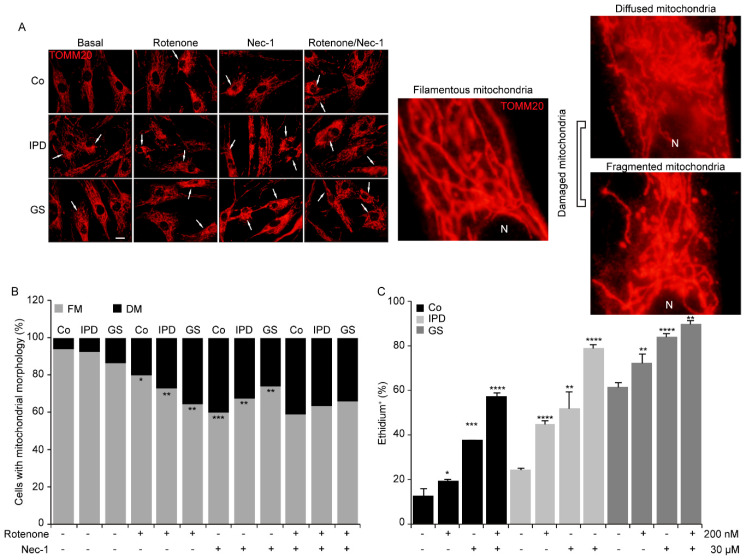
Necrostatin-1 alters mitochondrial morphology. (**A**–**C**) Human fibroblasts were treated with rotenone and/or necrostatin-1 (Nec-1) for 24 h. (**A**) Representative immunofluorescence images of cells immunolabeled with TOMM20. The scale bar represents 10 μm. The original magnification is ×40. N: nucleus. (**B**) Data are the percentages of cells with filamentous mitochondria (FM) and damaged mitochondria (DM) (* *p* < 0.05, ** *p* < 0.01, *** *p* < 0.001). (**C**) Fibroblasts were loaded with dihydroethidium (5 μM). The results represent the percentage ± SD of ethidium^+^ cells detected by flow cytometry (* *p* < 0.05, ** *p* < 0.01, *** *p* < 0.001, **** *p* < 0.001, compared to basal conditions), *n* = 10,000 events. Experiments were done two times.

The parkinsonian toxin rotenone causes mitochondrial fragmentation [23]. Thus, PD is related to abnormal mitochondrial morphology and oxidative stress [9]. To better understand the noxiousness of Nec-1, we questioned whether the mitochondrial injuries triggered by rotenone could be prevented with Nec-1 treatment. In this study, to evaluate mitochondrial morphology, we used TOMM20 staining and classified mitochondria into two groups: filamentous and damaged mitochondria. Damaged mitochondria were considered to be diffused or fragmented (Figure 4A, right panel). Then, we observed that the percentage of cells with damaged mitochondria increased remarkably in all cell lines with rotenone and/or Nec-1 treatments (Figure 4A,B). The inefficiency of Nec-1 in rotenone-treated cells was confirmed by the maintained level of damaged mitochondria and ROS generation (Figure 4C). In fact, the percentage of ethidium^+^ cells enhanced with rotenone was augmented with Nec-1. Moreover, Nec-1 alone affects mitochondrial morphology and increases ROS production, which could exacerbate the effect already caused by rotenone. Thus, we deduced that the toxicity of Nec-1 is related to mitochondrial dysfunction.

### 3.5. Necrostatin-1 Impairs Mitochondrial Clearance

**Figure 5:**
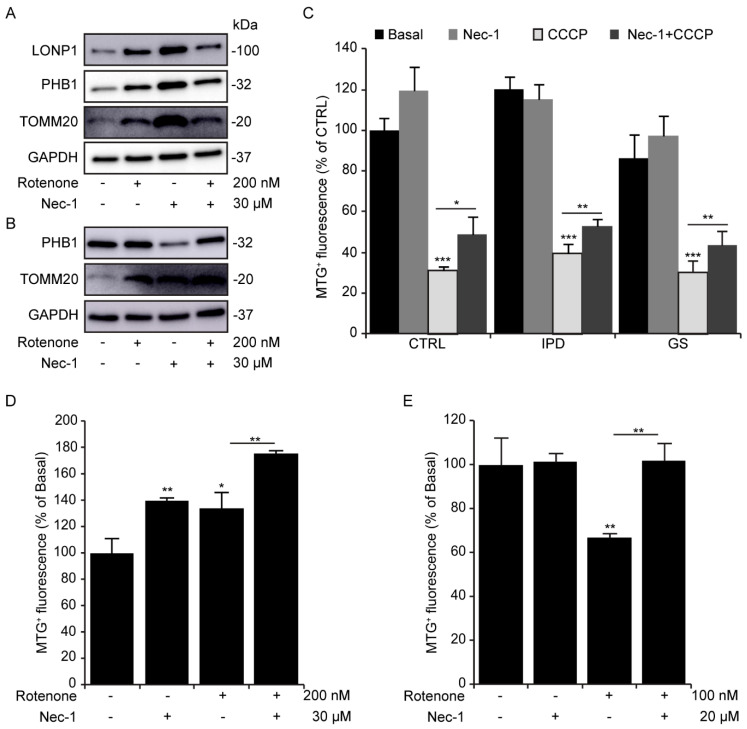
Necrostatin-1 increases the mitochondrial mass. (**A**,**B**) Human fibroblasts were treated with rotenone and/or necrostatin-1 (Nec-1) for 4 and 24 h. (**A**) The expression levels of LONP1, PHB1, and TOMM20 in human fibroblasts (HFs) after 4 h of treatment, (**B**) the expression levels of PHB1 and TOMM20 in HFs after 24 h of treatment. GAPDH is the loading control. (**C**) Human fibroblasts were treated with carbonyl cyanide 3-chlorophenylhydrazone (CCCP) and/or necrostatin-1 (Nec-1) for 24 h. Cells were loaded with 100 nM of MTG. The results represent the percentage ± SD of MitoTracker® Green (MTG^+^) cells detected by flow cytometry (* *p* < 0.05, ** *p* < 0.01, *** *p* < 0.001), *n* = 10,000 events. (**D**) Fibroblasts and (**E**) SH-SY5Y cells were treated with rotenone and/or necrostatin-1 (Nec-1) for 24 h and then loaded with 100 nM of MTG. The results represent the percentage ± SD of MTG^+^ cells detected by flow cytometry (** *p* < 0.01), *n* = 10,000 events. All experiments were done at least three times.

In fibroblasts treated four-hourly, rotenone as well as Nec-1 increased TOMM20 and PHB1 protein levels (Figure 5A). This increase was accompanied by an enhancement of LONP1, a mitochondrial protease involved in the PINK1-dependent mitophagy pathway [24]. After 24 h of rotenone and/or Nec-1 exposure, the expression level of TOMM20, but not PHB1, was consistent with that observed at 4 h (Figure 5B). We suggest that this could be due to inhibition of mitophagy, a selective mitochondrial clearance. In fact, Nec-1 has been reported to inhibit autophagosome formation in neurons treated with 6-OHDA [17]. To further elucidate the role of Nec-1 in mitochondrial degradation, we treated fibroblasts with CCCP (Figure 5C), a potent mitophagy inducer. Remarkably, treatment with Nec-1 alone did not lead to a significant increase in MTG^+^ fluorescence compared to Co cells, but it did inhibit CCCP-induced mitophagy in all cell lines. Although we did not observe mitophagy induction with rotenone in fibroblasts (Figure 5C), we did notice a significant reduction in MTG^+^ fluorescence in SH-SY5Y cells (Figure 5D), as previously reported [25], which was prevented by Nec-1. Overall, neuroblastoma cells were found to be more sensitive than fibroblasts to rotenone. Despite the effect of rotenone on mitochondrial turnover in both cell models, Nec-1 accumulated mitochondrial mass.

## 4. Discussion

Events that lead to neuronal death in PD are numerous and interconnected [26]. Thorough studies in PD cell death indicate that mitochondrial dysfunction, oxidative stress, and proteolysis deregulation together trigger cell death years before the appearance of the first clinical hallmarks [1]. Several cell death types can be found in postmortem brains of PD patients in *in vitro* and *in vivo* models of PD [26]. In this study, we explored necrotic programmed cell death (necroptosis) in primary fibroblasts from PD patients and used rotenone to model PD in cell culture. Necroptosis could be induced by ROS generation [27] and mitochondrial depolarization. Of note, the cells of PD patients displayed all of these previously cited characteristics [19]; however, necroptosis was not activated (Figure 1). Interestingly, rotenone induced necroptosis in SH-SY5Y cells (Figure 2C,D) and in primary fibroblasts (Figure 2E) by increasing the phosphorylation level of MLKL at ser358. If necroptosis was activated in postmortem brains of PD patients, it was not clearly activated in the primary fibroblasts. However, rotenone treatment made the cells of PD patients more susceptible to necroptosis activation. Rotenone-induced necroptosis was inhibited through the inactivation of RIP1, but other forms of cell death were activated or initiated in a RIP1-independent manner (Figure 3B,C). In fact, Nec-1 was found to inhibit RIP1 kinase activity and prevent the phosphorylation of MLKL and the plasma membrane rupture related to p-MLKL [14]. Even though necroptosis was inhibited in MLKL-deficient cells, the latter could undergo apoptosis [28]. In addition, 6-OHDA induced necroptosis in mesencephalic and cortical neurons. Nevertheless, inhibition through RIP1 or MLKL did not reduce nuclei condensation but did protect against axonal degeneration [16]. Most certainly, Nec-1 doses beneath 30 μM showed a neuroprotective effect on 6-OHDA-treated PC12 cells [17]. The doses used in our study promoted apoptotic and necrotic cell death in both rotenone-treated cell models. These data are consistent with Jie’s previous report [29].

**Figure 6:**
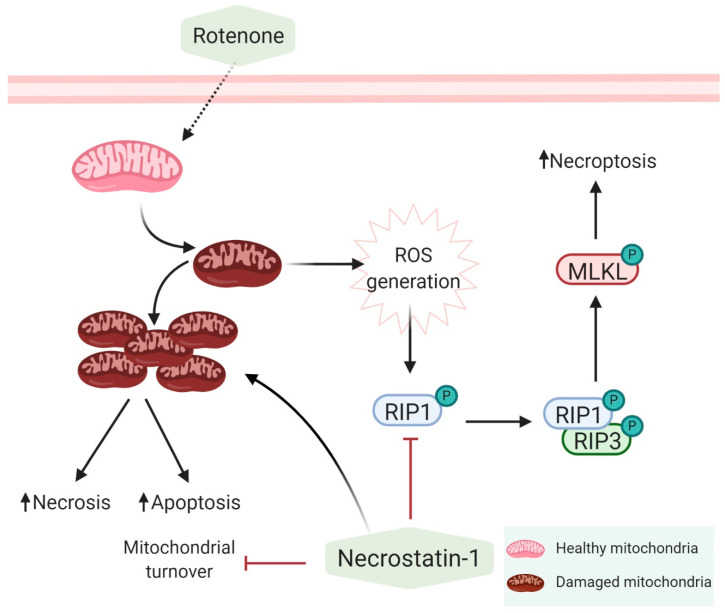
Mechanisms of action of rotenone and necrostatin-1. Rotenone inhibits the mitochondrial complex I and increases the production of reactive oxygen species (ROS) which, in turn, activates necroptosis via RIP1. Inhibition of RIP1 by Nec-1 inhibits necroptosis but does not reverse the effect of rotenone on complex I. In addition, Nec-1 inhibits mitophagy and exacerbates the toxicity of rotenone.

Rotenone is a mitochondrial complex I inhibitor [30], that acts as one of the necroptosis initiators. It has become crucial to determine why cells are still more susceptible to rotenone treatment despite the inhibition of necroptosis by Nec-1 at 20 μM in SH-SY5Y cells or 30 μM in fibroblasts. As a matter of fact, Nec-1 was found to decrease MMP (Figure 3A) and increase ROS production (Figure 3D or Figure 4C). We hypothesized that the inhibition of necroptosis by Nec-1 is mitochondria-independent and that Nec-1 failed to reverse rotenone-induced mitochondrial alterations and oxidative stress (Figure 4) by acting directly on the necrosome. It is noteworthy that Nec-1 downregulates autophagosome formation and lysosomal enzyme activity [17], illustrating that RIP1 kinase activity is not needed for autophagy inhibition [31]. Consistent with these reports, we think that Nec-1 inhibits mitophagy and fosters the accumulation of rotenone-induced mitochondrial damage which, in turn, induces oxidative stress, apoptosis, and necrosis. Swollen lysosomes (data not shown) may reveal the need to remove damaged mitochondria with Nec-1 treatment. Rotenone positively regulates mitophagy in SH-SY5Y cells via an externalized cardiolipin to the outer mitochondrial membrane, which binds to LC3 [25]. However, human fibroblasts showed lower sensitivity to rotenone (Figure 5D) than SH-SY5Y cells (Figure 5E), as reflected by the mitochondrial clearance response degree. Thus, the concentration of rotenone used was sufficient to trigger mitochondrial inclusion, but a higher concentration may be needed to remove accumulated mitochondria in fibroblasts. Nec-1 displayed different effects on internal and external mitochondrial membrane proteins at 24 h by Western blotting but increased the mitochondrial content with MTG staining by flow cytometry assay. Nec-1 was not shown to be effective for preventing rotenone-induced non-necroptotic cell death because of accumulation of mitochondrial damage and the activation of alternative mechanisms of cell death (Figure 6). Moreover, when Nec-1 showed limited effects on the survival of neurons, RIP3 ablation protected the cells treated with MPP^+^ against apoptosis [32]. However, divergent results suggest that the neuroprotective effect of RIP3 deletion is independent of apoptosis [15].

In fact, it was found that Nec-1 does not have the ability to reverse the inhibition of mitochondrial complex I by rotenone. Therefore, Nec-1 exerts a dual effect on cells through the inhibition of RIP1 kinase activity. By downregulating mitophagy, Nec-1 prevents the turnover of rotenone-injured mitochondria, maintaining the production of ROS and the activation of other non-necroptotic cell death pathways (Figure 6). In spite of necroptosis inhibition, mitophagy deregulation contributes to Nec-1 neurotoxicity. It would be interesting to investigate the use of a combined treatment of Nec-1 and/or autophagy modulators and even antioxidants to inhibit necroptosis and ROS production in PD cell models.

## Supplementary Materials

The following are available online at https://www.mdpi.com/2076-3921/9/6/524/s1, Figure S1: Expression level of the MLKL protein in primary human fibroblasts under basal condition.
